# The Unchampioned Nurse

**Published:** 1920-12-04

**Authors:** 


### THE UNCHAMPIONED NURSE.

I was once a member of a proprietary golf club—that is, a club run by the owner for profit. The members were permitted to manage their own affairs in all things except expenditure. If we wanted a new door-mat we had to petition the proprietor. He was really a good sort and not unreasonable. But he was canny, and one of the cleverest things he did was to come down to a council meeting and explain that we had for all practical purposes plenipotentiary powers, that he did not wish in any way to interfere with our arrangements, and that he would be perfectly satisfied if we appointed one of our number to act for him in the capacity of treasurer. We were all pleased, and we appointed our secretary, to whom we were paying a small salary, congratulating him that this would mean a substantial addition to his emoluments, and congratulating ourselves that our own colleague and chosen secretary would always be at hand to buy door-mats and other essentials.

It probably took us six months to realise that we had been sold. Our secretary became a positive miser on behalf of the other employer, and although he was always willing to admit the reasonableness of our demands, yet he had sheaves of arguments to advance why some little regard should be had for the interests of the proprietor, who had already expended so much. There were few, if any, refusals. Everything was done with great niceness, and our requests for finance were invariably held over for consideration "a few months hence when things are better."

It is a far cry to Thomas à Becket, Chancellor to King Henry the Second. It is no exaggeration to say that he was a bulwark of the throne. And King Henry made him Archbishop of Canterbury. A bishop who dissented from the policy of making this appointment sarcastically remarked at the termination of the ceremony that the King had worked a miracle in having that day turned a layman into an archbishop and a soldier into a saint. A few years later Henry, in a fit of fury, exclaimed in ungovernable fury, "Of the cowards who eat my bread, is there not one who will free me from this turbulent priest?"

### Serving two Masters?

Now one may be possessed of the very highest intentions; every action may be weighed with the most profound considerations of justice. Yet I think it will still be found true in the letter and in the spirit that no man can serve two masters. And I do not think it can be denied that the hospital board and the nursing staff of the hospital are in this sense two masters, with different and in some instances opposing interests. Let us drive the argument to its logical conclusion. Supposing that all the voluntary hospitals in England formed a Central Council, consisting of the chairmen of the various boards. How would it do to substitute this board for the Council of the College of Nursing? In other words, if Lord Knutsford, being Chairman of the "London," is a useful addition to the College Council, why not extend the principle?

The College of Nursing, with all its admirable features, is not a strong and virile institution. Lord Knutsford recognises the fact, and attributes it to its youth. The real cause, I beg to submit, is that many members of the Council, probably without being aware of the fact, actually hold watching briefs against the nurses while being their elected advocates. It is extremely difficult to conceive of a hospital matron being a whole-hearted nurses' champion, although there are in England a few who have achieved great things. But it is so frequently, if not always, a part of a matron's duty to safeguard hospital finance, that where expenditure is involved opposition is a natural attitude. It is, I contend, a very remarkable fact that during all this public controversy and discussion concerning voluntary hospitals and ways and means, the nurse and her just claims have been relegated to silence. There has not been even a half-hearted campaign conducted on her behalf.

### An Unwise Policy.

Of course, this is a policy as unwise as it is weak. The world continues to roll on its course, and the probationer problem becomes more difficult. I see that the London County Council are suggesting to teachers of their evening institutes for women that girls should be systematically taught, amongst other things, the price and value of their work. Everywhere, save in the nursing world, women are travelling on the upward way. They have their unions and their champions. The North Islington Maternity Centre and School for Mothers, Holloway Road, of which H.R.H. Princess Christian is patroness, is making a departure which, if it is progressive and admirable in one respect, is a blow to the prospects of trained nurses. Girls at the age of fourteen and sixteen are being taken for a four years' or two years' preliminary course, during which, besides continuing their general education at evening classes, they receive training in infant-care, handling and dressing babies, sewing infants' garments, and attending lectures on physiology, hygiene, and first aid. *The Times*, commenting on this, points out that "by the age of eighteen they are ready for taking up one of the several careers connected with the care of the young or the sick, as nurses, nursery-school teachers, trained nursery governesses, or health visitors." And meantime the present generation of nurses drift along, quiescent, while the College Council is quietly getting out of its youthful helplessness.

Can any serve two masters? IERNE.

